# Lower serum nicotinamide N-methyltransferase levels in patients with bipolar disorder during acute episodes compared to healthy controls: a cross-sectional study

**DOI:** 10.1186/s12888-020-2461-4

**Published:** 2020-01-30

**Authors:** Qing Hu, Farong Liu, Luyin Yang, Zanxi Fang, Jue He, Wenqiang Wang, Pan You

**Affiliations:** 1 Xiamen Xianyue Hospital, 399# Xianyue Road, Xiamen, 361012 China; 2 The First Affiliated Hospital of Xiamen University, Xiamen, China; 3 Arts College of Xiamen University, Xiamen, China; 4 Zhongshan Hospital, School of Medicine, Xiamen University, Xiamen, China

**Keywords:** Nicotinamide-N-methyltransferase, Bipolar mania, Enzyme-linked immunosorbent assay, Lipid metabolism, Serum level

## Abstract

**Background:**

Nicotinamide N-methyltransferase (NNMT) has been implicated in the pathogenesis of neuropsychiatric diseases. Bipolar disorder (BD) is associated with metabolic abnormalities and NNMT regulates energy metabolism and may also exert a causal role in metabolic disorders. The present study aimed to determine serum NNMT levels in patients with BD and compared the results with that of healthy controls, to explore the correlation between NNMT and clinical and metabolic characteristics.

**Methods:**

The NNMT levels of 80 patients having a manic episode of BD and 65 non-psychiatric control individuals were measured using enzyme-linked immunosorbent assay. Metabolic parameters were evaluated using standard laboratory methods.

**Results:**

The serum NNMT levels of bipolar mania patients were significantly lower than that of non-psychiatric controls. Furthermore, the serum levels of NNMT were found to be negatively correlated with Young Mania Rating Scale (YMRS) scores and the duration of the illness. Moreover, lower NNMT serum levels were found in patients with a history of antipsychotic medication and dyslipidemia. Our results also demonstrated the different patterns of correlation that exist between the study groups. Serum NNMT levels were found to be negatively correlated with triglyceride, cholesterol, and apolipoprotein B levels in the BD group, while the same was found to be negatively associated only with high-density lipoprotein cholesterol in the control group.

**Conclusions:**

These findings support the suggestion that lower NNMT serum levels are significantly associated with BD and that serum NNMT has the potential to regulate lipid metabolism in BD patients.

## Background

Bipolar disorder (BD) is a lifelong episodic disorder characterized by fluctuations in mood state and energy [1]. BD affects more than 1% of the world’s population and represents one of the main causes of disability among young people [2]. Health problems, including but not limited to, cardiovascular disorders, hyperlipidemia and hyperglycemia are highly prevalent and occur relatively earlier in individuals with BD than the general population [1]. For instance, there is up to a three times increased risk of type 2 diabetes mellitus in patients with BD compared to the general population [3–5]. The prevalence of metabolic syndrome is 30% higher in bipolar patients than in general population [6].

The exact etiology leading to the onset of BD episodes are still largely unknown [7]. Recently, special attention has been focused on one-carbon metabolism, as it may play a central role in neuropsychiatric diseases. One-carbon metabolism includes three metabolic processes:folate metabolism, homocysteine remethylation cycle, and sulfur transfer pathway [8]. It is associated with many biochemical reactions (such as methylation processes) and plays an important role in certain essential metabolisms (such as the synthesis of amino acids and peptides). Imbalances of one-carbon metabolism have been reported to be associated with some neuropsychiatric diseases (such as schizophrenia, Alzheimer’s disease, and BD) and may be involved in cardiovascular disorders [9–11]. Nicotinamide-N-methyltransferase (NNMT) is an important enzyme that is involved in one-carbon metabolism. Human NNMT was first identified in the liver [12] and was later found to be expressed in a variety of tissues including brain and other nervous system tissues [13]. NNMT plays a crucial role by modulating nicotinamide and some pyridine derivatives methylation, which has been thought to have an important role in the biochemical basis of neuropsychiatry [8, 14].

Emerging evidence has found a correlation between NNMT and many neuropsychiatric diseases. A recent study showed that neuronal NNMT expression in *C. elegans* affects behavior, neurodegeneration, and life expectancy by controlling neuronal autophagy, which may increase the risk of developing Parkinson’s disease (PD) and schizophrenia in humans [15]. High expression of NNMT has been found in the post-mortem cerebella of Parkinson’s patients [16], and lower NNMT mRNA expression has been detected in the post-mortem frontal cortex of schizophrenia (SZ) patients, compared with that of control individuals [17]. Two genetic association studies found that a specific mutant in the NNMT gene is significantly associated with the occurrence of BD and SZ [18, 19]. NNMT regulates energy metabolism and may also exert a causal role in metabolic disorders. Elevated levels of me-NNMT, an indicator of NNMT activity, were found to be correlated with insulin resistance, and increased adipose NNMT expression has been observed in type 2 diabetic patients [20]. In studies on mice, knockdown of NNMT in liver and adipose tissue caused an increase in energy consumption, and attenuated diet-related obesity and worsening of metabolic disorders [21]. Studies have also demonstrated that liver NNMT expression is negatively correlated with multiple metabolic parameters, including glucose (Glu), cholesterol (CHO), low-density lipoprotein (LDL), High-density lipoprotein (HDL), and triglycerides (TG) levels [22].

In light of these observations, we hypothesized that NNMT may also play a role in the pathophysiology of BD and may be associated with the aberrant metabolism in BD patients. In other words, NNMT may be a biomarker for BD or a therapeutic target for metabolic disorders in people with psychiatric illness. In this study, serum NNMT levels and its correlation with clinical and metabolic characteristics were investigated in individuals with acute mania BD.

## Methods

### Participants

Eighty patients (31 females, 49 males) having an acute manic BD episode were anonymously recruited to the study, before receiving systemic treatment at the Xiamen Xianyue Hospital, Fujian, China, after informed consent was obtained. Diagnosis of BD for each patient was made based on guidelines of the International Classification of Diseases 10th Revision (ICD-10) by two experienced psychiatrists. The YMRS [23] was used to assess the severity of manic symptoms. The healthy volunteers control group was screened for psychiatric disorders using the Chinese Minnesota Multiphasic Personality Inventory-2 (MMPI-2) [24]. The inclusion criteria were that each subscale has a score of less than 60. Finally, 65 (31 females, 34 males) age-and sex-matched healthy volunteers with no history and family history of major psychiatric disorders, intellectual disability, dementiaor the use of psychotropic substances were selected as controls. Exclusion criteria included the following: presence of tumors, pregnancy or breastfeeding. The study was approved by the ethics committee of Xiamen Xianyue Hospital, Fujian, China.

### Clinical and biochemical measurements

Overnight fasting venous blood samples were collected in anticoagulant-free tubes from all experimental subjects after they had fasted overnight. After one-hour of incubation at room temperature, the serum was separated by centrifuging the samples for 10 min at 4000 rpm. Subsequently, the samples were aliquoted and stored at − 80 °C for the measurement of NNMT. The serum levels of glucose and lipids including TG, CHO, HDL-c, low-density lipoprotein cholesterol (LDL-c) and apolipoprotein A (apo A), apo B were measured using standard laboratory methods. Serum NNMT levels were measured using ELISA kits, according to the manufacturer’s instructions (Human NNMT ELISA KIT, Cat#JL19694, Jianglai Biotechnology Co., Ltd.). Optical density was measured using a microplate luminometer at 450 nm, and sample concentrations were calculated using standard curves and are expressed in U/L.

Psychiatric/medical history and demographic data of the patients were documented by an experienced psychiatrist. Systolic blood pressure (SBP) and Diastolic blood pressure (DBP) of the subjects was measured by the nurse before blood collection. Body weight and height were measured by a nurse. BMI was calculated using the formula: BMI = weight (kg)/ [height (m)]^2^. Obesity was defined as a BMI ≥28 kg/m^2^, based on Chinese criteria [25]. Dyslipidemia was defined based on abnormal laboratory parameters [26].

### Statistical analysis

The normality of the data was determined using Kolmogorov-Smirnov and Shapiro–Wilk tests, and Levene’s test was used to calculate the homogeneity of variance. Differences of normally distributed variables (presented as mean ± SD) between groups were examined using student’s unpaired *t* test, while non-normal distribution variables (presented as median with interquartile range) were compared using Mann-Whitney *U* tests. Adjustment for confounding factors including age, gender, BMI, age of onset, illness duration, total hospitalization, education, a family history of psychiatric illness was conducted using multiple stepwise regression analysis. Linear regression analysis was used to examine the correlations between serum NNMT levels and metabolic parameters, after adjustment for age, gender, and BMI.

Statistical analyses were conducted using IBM SPSS Statistics version 21.0 software (Chicago Inc., USA), and a *p* value of < 0.05 was considered to be statistically significant.

## Results

### Sample characteristics

64 (44.1%) of the 145 study subjects (mean age 35.3 ± 13.2 years) were females. The mean BMI was 24.29 kg/m^2^ (SD 3.42) and 19 (13.1%) participants were obese (BMI ≥ 28 kg/m^2^), 55 (37.9%) had dyslipidemia, and 11 (7.6%) had impaired glucose metabolism. Detailed metabolic characteristics of the BD patients and control individuals are presented in Table 1. No significant differences in TG, HDL-c, LDL-c, apo B, and fasting blood glucose (FBG) levels were observed between control and BD individuals, but SBP and DBP in BD patients were higher than in healthy controls, and BD patients had significantly higher levels of serum CHO and apo A (all *p* < 0.05). The mean BMI and frequency of obesity seemed to be higher in individuals with BD, but the differences were not statistically significant (*p* > 0.05).

**Table 1:** Demographic and clinical characteristics of the study participants at inclusion

	Healthy controls (*n* = 65)	BD patients (*n* = 80)	*p*^†^
Age (years)	34 (25, 46)	31 (24, 45)	–
Sex (M/F)	34/31	49/31	–
Metabolic parameters			
BMI (kg/m^2^)	23.03 (21.76, 24.53)	23.98 (22.29, 26.79)	0.064
SBP (mmHg)	120 (112, 135)	126 (120, 142)	0.031^*^
DBP (mmHg)	80 (70, 85)	85 (71, 88)	0.049^*^
TG (mmol/L)	1.03 (0.71, 1.76)	1.42 (0.92, 1.83)	0.105
CHO (mmol/L)	4.31 (3.83, 5.42)	4.70 (4.33, 5.53)	0.026^*^
HDL-c (mmol/L)	1.24 (1.05, 1.47)	1.29 (1.09, 1.48)	0.618
LDL-c (mmol/L)	3.05 (2.62, 3.65)	3.14 (2.85, 3.64)	0.24
apo A(g/L)	1.37 (1.23, 1.49)	1.51 (1.35, 1.58)	0.004^**^
apo B(g/L)	0.92 (0.77, 1.12)	0.88 (0.75, 1.02)	0.529
FBG (mmol/L)	5.06 (4.47, 5.34)	4.89 (4.49, 5.67)	0.808
Comorbidities			
Obesity, n (%)	6 (9.2%)	13 (16.3%)	0.159
Dyslipidemia, n (%)	25 (38.5%)	30 (37.5%)	0.316
IGM, n (%)	5 (7.7%)	6 (7.6%)	0.603
Medications			
SGA+ MS, n (%)	–	23 (28.8%)	–
SGA+ AED, n (%)	–	22 (27.5%)	–
SGA+ MS+ AED, n (%)	–	12 (15%)	–
Drug-naive, n (%)	–	23 (28.8%)	–

Data are presented as mean ± SD, median (interquartile range), or n (%). ^†^*p*-values of Mann-Whitney *U* test, Student’s unpaired *t* test or *χ*^2^

^∗^*p <* 0.05, ^∗∗^*p <* 0.01

### Serum NNMT level analysis in the samples

**Fig. 1:**
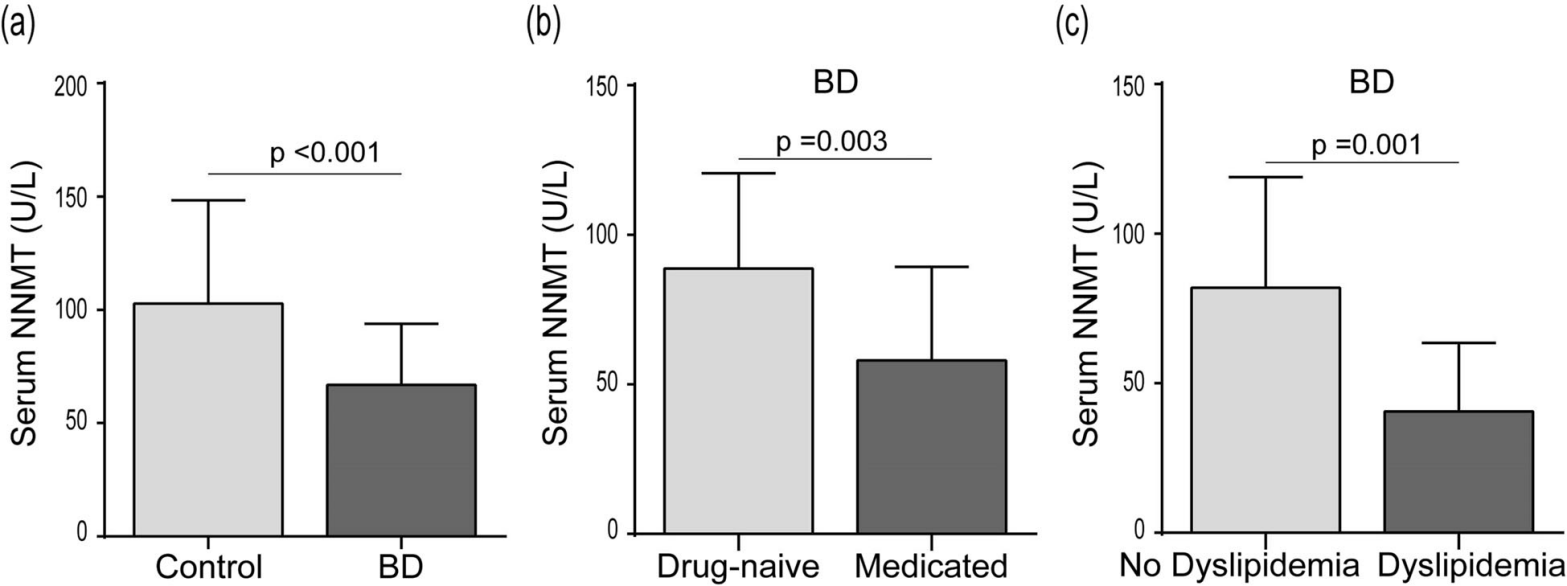
Serum NNMT levels (**a**) in Control group VS BD group, and in patients stratified according to (**b**) whether there is a history of psychotropic use and (**c**) the presence of dyslipidemia

For this study cohort, NNMT levels ranged from 5.436 to 299.223 U/L, and the Mann–Whitney *U* test indicated that the serum NNMT levels BD patients were significantly lower (54.00 U/L [21.74, 96.14]) than that of healthy controls (85.36 U/L [48.82, 123.38]) (*p* < 0.01) (Fig. 1a). Moreover, in 57 (71.3%) patients with recurrent bipolar mania and a history of psychotropic medication (Table 1), NNMT levels were found to be significantly lower than first-episode and/or drug-naive patients (Fig. 1b, *p* = 0.003). However, the results showed no difference in levels of NNMT among patients using different types of psychotropic drugs (Additional file 1: Table S1. Kruskal-Wallis test, *p* = 0.280).

### Correlation between serum NNMT levels and the clinical characteristics of individuals with BD

As shown in Table 2, confounding factors, including age, gender, BMI, age of onset, illness duration, total hospitalization, education, family history of psychiatric illness was found to not be associated with serum NNMT levels. However, it is worthy to note that YMRS scores (*r* = − 0.390, *p* < 0.001) and duration of the illness (*r* = − 0.277, *p* = 0.013) were found to be negatively correlated with serum NNMT levels.

**Table 2:** Clinical data for BD patients and associations with serum NNMT levels

	Values	r	*p*^‡^
Age (years)	31 (24, 45)	−0.018	0.876
Sex (M/F)	49/31	−0.102	0.370
BMI	23.98 (22.29, 26.79)	0.029	0.797
YMRS total score	35.83 ± 6.42	−0.390	< 0.001^∗∗^
Age of onset (years)	22 (16, 29)	0.124	0.075
Duration of illness (years)	6 (1.5, 15)	−0.277	0.013^*^
Total hospitalization (n)	3 (2, 6)	0.026	0.819
Education (years)	9 (9,12)	−0.2	0.085
Family history of psychiatric illness, n (%)	13 (16.3%)	0.026	0.411

Data are presented as mean ± SD, median (interquartile range), or n (%). ^‡^*p*-values of multiple stepwise regression analysis, after adjustment for confounding factors

^∗^*p <* 0.05, ^∗∗^*p <* 0.01

### Correlation between serum NNMT and metabolic profile

After adjustment for age, gender, and BMI, correlations between serum NNMT levels and laboratory metabolic parameters were found in BD patients and control individuals. These correlations observed were different in the two groups (as shown in Table 3). Only the level of HDL-c was found to be negatively associated with serum NNMT levels in the control group (*r* = − 0.303; *p* = 0.014), while no correlation between the HDL-c and NNMT levels (*p* = 0.887) were found in the BD patient group, which instead showed a negative correlation between metabolic parameters, such as TG, CHO, and apo B levels and serum NNMT levels. We wondered whether these differences regarding metabolic parameters between BD patients and control individuals could be partially explained by the differences in serum NNMT levels. Subgroup analyses, based on the presence of dyslipidemia or obesity showed that lower NNMT levels in patients with dyslipidemia in the BD subgroups (Fig. 1c, *p* = 0.001) but not in the control subgroups (*p* = 0.183), whereas obesity did not have an effect either of the subgroups (all *p* > 0.05).

**Table 3:** Correlation between serum NNMT levels and metabolic parameters in healthy controls and BD individuals

	Healthy controls		BD patients	
	*r*^†^	*p*^§^	*r*^‡^	*p*^¶^
SBP	−0.050	0.372	−0.142	0.103
DBP	0.109	0.401	−0.056	0.619
TG	−0.15	0.232	**−0.447**	**< 0.001**^∗∗^
CHO	−0.186	0.138	**−0.527**	**< 0.001**^∗∗^
HDL-c	**−0.303**	**0.014**^∗^	−0.016	0.887
LDL-c	−0.175	0.164	−0.207	0.065
apo A	0.116	0.359	−0.116	0.304
apo B	−0.17	0.176	**−0.536**	**< 0.001**^∗∗^
FBG	0.072	0.568	−0.082	0.47

^**§**/**¶**^*p*-values of linear regression analysis, adjusted for age, gender and BMI

^∗^*p <* 0.05, ^∗∗^*p <* 0.01

## Discussion

In the present study, we explored the serum levels of NNMT in BD patients. Results showed that the serum NNMT levels of BD manic patients were significantly lower, compared with that of age and sex matched controls. Furthermore, the serum levels of NNMT were found to be negatively correlated with YMRS scores and the duration of the illness. Moreover, lower NNMT serum levels were found in patients with a history of antipsychotic medication and dyslipidemia. Interestingly, the correlations between NNMT and metabolic parameters were different in the BD patient group, compared with that found in the control group, indicating a correlation between aberrant NNMT and metabolic dysregulation that is only prevalent in the BD population.

NNMT is highly expressed in human liver and is considered to be a cytoplasmic protein [12], Human NNMT was also found to be expressed in brain and other nervous system tissues [13], and a growing body of evidence has found a correlation between NNMT and many neuropsychiatric diseases. In the present study, we detected NNMT levels using ELISA on BD patients and healthy control individuals without mental illness. Results showed that the serum level of this protein in bipolar mania patients was significantly lower than that of healthy controls. Studies have reported that NNMT is expressed in the brain [13], and that the level of NNMT protein in the cerebellum and cerebrospinal fluid of Parkinson’s patients was significantly higher than that of healthy controls [27, 28]. In a postmortem frontal cortex study of 26 participants, NNMT mRNA expression was found to be significantly lower in schizophrenia patients, compared with that of control individuals [17]. However, the mechanism by which NNMT protein is transported across the blood–brain barrier is not yet known. There are no reports of positive correlations between brain and serum NNMT levels. Therefore, further research is needed to elucidate the potential correlation between circulating NNMT and neuronal expression levels, which may better explain the role of abnormal levels of serum NNMT observed patients with neuronal disorders.

Studies have shown that dysregulation of one-carbon metabolism is closely linked with functional deficiency and the treatment effect of BD [29]. For example, high homocysteine levels were found to be harmful to neurons and correlated with poor functioning bipolar manic patients [30]; folate deficiency was found to be associated with greater symptom severity and worse treatment responses in bipolar depressive patients. NNMT is one of the key enzymes involved in one-carbon metabolism and it regulates the generation of homocysteine. The correlation between NNMT and BD was previously reported in a genotyping study, in which a variant of the NNMT gene was found to be a genetic risk factor for BD [18]. We found a significant correlation between lower serum NNMT levels and YMRS scores, as well as the duration of the illness, which indicates its involvement in the etiology of BD. Although the exact pathogenesis and molecular mechanism of these relationships are not yet clearly known, it can be assumed that abnormal levels of NNMT may influence the epigenetics and/or the blood levels of homocysteine, and nicotinamide in BD patients.

Interestingly, our study also identified that the correlation between serum NNMT levels and metabolic parameters is different in BD from that of the correlation found with control individuals, suggesting that lower NNMT levels may be associated with certain confounding factors. The impact of one-carbon metabolism on metabolic dysregulation has been demonstrated previously [31]. In recent years, NNMT has also emerged as a novel metabolic regulator. It has been demonstrated that liver NNMT expression is inversely correlated with multiple metabolic parameters [22], and that NNMT knockdown protects mice from adiposity and its deleterious metabolic consequences [21]. In line with these findings, serum NNMT levels were found to negatively correlate with several lipid parameters in our study population. NNMT expression was found to be negatively correlated only with HDL-c in the control group, but was found to be negatively correlated with TG, CHO, and apo B in the BD group and a decrease in NNMT expression was found in patients with dyslipidemia and in those with a history of psychotropic medication. That was to say, lower levels of serum NNMT in bipolar mania patients was associated with higher serum lipid levels and was related to the use of psychotropic drugs. Patients with psychiatric disorders, including BD subjects, are more prone to metabolic disorders than the general population [1]. It has been reported that antipsychotic drugs may lead to higher lipid levels and increase the risk of metabolic disorders in psychotic patients [32]. Therefore, we tend to believe that these disparate correlations may render a higher metabolic risk in patients with BD.

In this study, we analyzed the statistical power = 0.90088 (Additional file 2: Table S2), which indicates that the sample sizes of the groups are appropriate. However, there are several limitations in our study. For example, parameters, such as liver function, insulin level, or drug dose, which may be closely associated with NNMT, were not included in this study. Hence, future studies that take into consideration detailed clinic characteristics will be helpful in clarifying the correlation between NNMT and metabolic dysregulation in BD patients. Previous reports have indicated that NNMT activity plays a causal role in metabolic disorders and cardiovascular diseases in humans [33]. However, in this study, only the serum level of NNMT was measured, leaving the magnitude of its activity unknown, which should be taken into consideration in future studies.

## Conclusions

In summary, our study shows that serum NNMT levels are lower in BD-manic patients, compared with that of non-psychiatric control individuals, and that the lower levels are negatively correlated with YMRS scores and the duration of the illness. The correlations between NNMT and metabolic parameters observed in the BD group are different from those observed in the control group. We also found significantly lower serum NNMT levels in BD patients with dyslipidemia than those without dyslipidemia. Moreover, NNMT levels were found to be lower in BD patients with a history of psychotropic medication than in drug-naive patients. Our results suggest that alterations in the serum NNMT level may play an important role in the pathology and metabolic dysregulation of BD individuals.

## Supplementary information

**Additional file 1: Table S1.** History of psychotropic medication in patients with recurrent bipolar mania and expression levels of serum NNMT between groups.

**Additional file 2: Table S2.** Numeric Results for Two-Sample T-Test Allowing Unequal Variance Alternative Hypothesis: H1: δ = μ1 - μ2 ≠ 0.

## Data Availability

The data used during the current study are available from the corresponding author on reasonable request, and the data are anonymized.

## Abbreviations

AED: Antiepileptic drugs
apo B: apolipoproteinB
apoA: apolipoproteinA
BD: Bipolar disorder
BMI: Body mass index
CHO: Cholesterol
DBP: Diastolic blood pressure
F: Female
FBG: Fasting blood glucose
Glu: Glucose
HDL-c: High-density lipoprotein cholesterol
ICD-10: International Classification of Diseases 10th Revision
IGM: Impaired glucose metabolism
LDL-c: Low-density lipoprotein cholesterol
M: Male
MS: Metabolic syndrome
MS: Mood stabilizer
NNMT: Nicotinamide N-methyltransferase
PD: Parkinson’s disease
SBP: Systolic blood pressure
SGA: Second-generation antipsychotics
SZ: Schizophrenia
TG: Triglyceride
YMRS: Young Mania Rating Scale
