# Supplementary material for: Lower serum nicotinamide N-methyltransferase levels in patients with bipolar disorder during acute episodes compared to healthy controls: a cross-sectional study
Source: BMC Psychiatry. 2020 Jan 30;20:33. doi: 10.1186/s12888-020-2461-4 (PMC6990555; doi:10.1186/s12888-020-2461-4)
Supplement: Supplementary file 1 — Additional file 1: Table S1. History of psychotropic medication in patients with recurrent bipolar mania and expression levels of serum NNMT between groups. [file 12888_2020_2461_MOESM1_ESM.docx]

**Additional file 1**

**Table S1:** **History of psychotropic medication in patients with recurrent bipolar mania and expression levels of serum NNMT between groups.** Kruskal-Wallis test were used for comparisons between groups.

| **Psychotropic medication**  **n (%)** | SGA & lithium  23 (40.4) | SGA & Antiepileptic drugs  22(38.6) | SGA & lithium & Antiepileptic drugs  12(21.1) | ***Χ²*** | ***p*** |
| --- | --- | --- | --- | --- | --- |
| **NNMT (U/L)** | 46.85±14.64 | 53.19±9.20 | 56.56±7.06 | **2.547** | **0.280** |

**Abbreviation:** SGA: Second generation atypical antipsychotics
