# Supplementary material for: Lower serum nicotinamide N-methyltransferase levels in patients with bipolar disorder during acute episodes compared to healthy controls: a cross-sectional study
Source: BMC Psychiatry. 2020 Jan 30;20:33. doi: 10.1186/s12888-020-2461-4 (PMC6990555; doi:10.1186/s12888-020-2461-4)
Supplement: Supplementary file 2 — Additional file 2: Table S2. Numeric Results for Two-Sample T-Test Allowing Unequal Variance Alternative Hypothesis: H1: δ = μ1 - μ2 ≠ 0. [file 12888_2020_2461_MOESM2_ESM.docx]

**Additional file 2**

**Table S2:** **Numeric Results for Two-Sample T-Test Allowing Unequal Variance**

**Alternative Hypothesis: H1: δ = μ1 - μ2 ≠ 0**

| Power | Control (N1) | Patients (N2) | Total (N) | μ1 | μ2 | δ | σ1 | σ2 | Alpha |
| --- | --- | --- | --- | --- | --- | --- | --- | --- | --- |
| **0.90088** | 65 | 80 | 145 | 102.8 | 66.9 | 35.9 | 72.4 | 56.6 | 0.050 |

Two-Sample T-Tests using PASS15.0
